# Preoperative Maximum Standardized Uptake Value Emphasized in Explainable Machine Learning Model for Predicting the Risk of Recurrence in Resected Non–Small Cell Lung Cancer

**DOI:** 10.1200/CCI-24-00194

**Published:** 2025-03-05

**Authors:** Takafumi Iguchi, Kensuke Kojima, Daiki Hayashi, Toshiteru Tokunaga, Kyoichi Okishio, Hyungeun Yoon

**Affiliations:** ^1^ Department of General Thoracic Surgery, NHO Kinki Chuo Chest Medical Center, Osaka, Japan; ^2^ Clinical Research Center, NHO Kinki Chuo Chest Medical Center, Osaka, Japan; ^3^ Department of Thoracic Oncology, NHO Kinki Chuo Chest Medical Center, Osaka, Japan

## Abstract

**PURPOSE:**

To comprehensively analyze the association between preoperative maximum standardized uptake value (SUV_max_) on 18F-fluorodeoxyglucose positron emission tomography-computed tomography and postoperative recurrence in resected non–small cell lung cancer (NSCLC) using machine learning (ML) and statistical approaches.

**PATIENTS AND METHODS:**

This retrospective study included 643 patients who had undergone NSCLC resection. ML models (random forest, gradient boosting, extreme gradient boosting, and AdaBoost) and a random survival forest model were developed to predict postoperative recurrence. Model performance was evaluated using the receiver operating characteristic (ROC) AUC and concordance index (C-index). Shapley additive explanations (SHAP) and partial dependence plots (PDPs) were used to interpret model predictions and quantify feature importance. The relationship between SUV_max_ and recurrence risk was evaluated by using a multivariable Cox proportional hazards model.

**RESULTS:**

The random forest model showed the highest predictive performance (ROC AUC, 0.90; 95% CI, 0.86 to 0.97). The SHAP analysis identified SUV_max_ as an important predictor. The PDP analysis showed a nonlinear relationship between SUV_max_ and recurrence risk, with a sharp increase at SUV_max_ 2-5. The random survival forest model achieved a C-index of 0.82. A permutation importance analysis identified SUV_max_ as the most important feature. In the Cox model, increased SUV_max_ was associated with a higher risk of recurrence (adjusted hazard ratio, 1.03 [95% CI, 1.00 to 1.06]).

**CONCLUSION:**

Preoperative SUV_max_ showed significant predictive value for postoperative recurrence after NSCLC resection. The nonlinear relationship between SUV_max_ and recurrence risk, with a sharp increase at relatively low SUV_max_ values, suggests its potential as a sensitive biomarker for early identification of high-risk patients. This may contribute to more precise assessments of the risk of recurrence and personalized treatment strategies for NSCLC.

## INTRODUCTION

Cancer remains a significant global health burden despite advances in prevention, early detection, and treatment. The American Cancer Society projects over 2 million new cancer cases and 611,720 cancer deaths in the United States by 2024.^1^ Among these, non–small cell lung cancer (NSCLC) continues to be among the most lethal malignancies worldwide.^2^ Despite surgical resection being the standard curative treatment for NSCLC, high recurrence rates (15%-38%) demonstrate the need for improved postoperative recurrence prediction.^3^ Preoperative identification of high-risk patients could optimize neoadjuvant and adjuvant therapies and surveillance. 18F-fluorodeoxyglucose positron emission tomography-computed tomography (PET-CT) is widely used in staging and assessing the treatment response of NSCLC. The maximum standardized uptake value (SUV_max_), reflecting tumor glucose metabolism, has been associated with tumor aggressiveness and prognosis.^4-6^ However, the clinical significance of SUV_max_ in predicting the recurrence of NSCLC remains controversial, limiting its practical application.^7,8^ Few studies have examined the continuous relationship between SUV_max_ and the risk of recurrence without arbitrary cutoff values. Despite the promise of machine learning (ML) in the health care prognostics, its application to elucidate the importance of SUV_max_ in predicting recurrence of NSCLC remains limited.^9^ This study aimed to comprehensively analyze the association between preoperative SUV_max_ and postoperative recurrence in NSCLC using ML and statistical approaches. By evaluating SUV_max_ as a continuous variable, we sought to uncover complex interactions potentially missed by conventional methods with the aim of achieving more precise stratification of the risk of recurrence. Our findings may refine risk assessment, advance personalized treatment strategies, and enhance clinical decision making in NSCLC management through data science.

**CONTEXT**

**Key Objective**
How does preoperative maximum standardized uptake value (SUV_max_) on 18F-fluorodeoxyglucose positron emission tomography-computed tomography predict postoperative recurrence in resected non–small cell lung cancer (NSCLC) when analyzed using advanced machine learning techniques alongside traditional statistical methods?
**Knowledge Generated**
Random forest models identified SUV_max_ as a key predictor of recurrence, revealing a nonlinear relationship with sharply increasing risk between SUV_max_ values of 2-5. Random survival forest analysis ranked SUV_max_ as the most important feature for predicting recurrence-free survival.
**Relevance**
The findings in this work could help in improving cancer treatment planning, care delivery, and outcomes in patients with NSCLC.

## PATIENTS AND METHODS

### Patients

This retrospective study included 643 patients who underwent R0 resection of NSCLC at the NHO Kinki Chuo Chest Medical Center (KCMC) between April 2017 and June 2022. Histopathologic diagnoses followed the 2015 WHO classification.^10^ Clinicopathologic data, including SUV_max_, age, sex, preoperative neutrophil-to-lymphocyte ratio (NLR), pathologic stage (American Joint Committee eighth edition), histologic type, vascular invasion (v) and lymphovascular invasion (Ly), adjuvant chemotherapy, the expression of PD-L1 (assessed by 22C3 pharmDx kit), and postoperative recurrence-free survival (RFS), were collected. Eligible patients received platinum-based adjuvant chemotherapy according to the relevant Japanese guidelines. The KCMC Ethics Committee approved the study (No. 2023-73) and waived the need for informed consent owing to the retrospective nature of this study. All methods adhered to the relevant guidelines and regulations.

### RFS

The primary end point was diagnosis of postoperative recurrence. RFS was measured from surgical resection until clinically confirmed recurrence. Postoperative surveillance included three to six monthly blood tests and radiographic examinations. Suspected recurrence prompted further evaluation (eg, brain magnetic resonance imaging, contrast-enhanced CT, PET, and biopsy). Recurrence was diagnosed through a multidisciplinary review by thoracic surgeons, medical oncologists, pathologists, and radiologists.

### ML

We used four classification algorithms (random forest, gradient boosting, extreme gradient boosting [XGBoost], and AdaBoost) and a random survival forest model to predict postoperative recurrence in patients with NSCLC. Input variables were chosen on the basis of previously reported factors associated with postoperative recurrence, including SUV_max_, RFS, pathologic stage, v,^11^ Ly,^12^ histologic type, PD-L1 expression,^13^ NLR,^14^ adjuvant chemotherapy status,^15^ age, and sex. The data set was partitioned into training (80%), validation (10%), and testing (10%) data sets to mitigate overfitting. The models were constructed using Python libraries and their hyperparameters were optimized using Bayesian optimization, which was implemented using the Optuna library. For each algorithm, 100 optimization trials were performed using Optuna, and the hyperparameter configuration that yielded the best performance was selected as the optimal setting (Data Supplement, Table S1). Model performance was evaluated using accuracy, F1 score, Brier score, receiver operating characteristic (ROC) AUC, and precision-recall (PR) AUC for the classification models, and concordance index (C-index) for the random survival forest model. The best-performing model was selected for further analysis. Additionally, we used bootstrap evaluation with 1,000 iterations to further validate the performance of the best-performing model. Out-of-bag (OOB) samples were used to compute performance metrics, providing a robust estimate of model generalizability. The Shapley additive explanations (SHAP) were used to interpret model predictions and quantify feature importance, while partial dependence plots (PDPs) were used to investigate the influence of SUV_max_ on model predictions.^16,17^ In addition, we performed 10-fold cross-validation to assess the robustness of our findings, particularly focusing on the relationship between SUV_max_ and partial dependence in predicting postoperative recurrence. For the random survival forest model, permutation importance was used to evaluate feature importance by measuring the decrease in C-index when permuting feature values.^18^ Python version 3.10.12 was used for ML model development and interpretation, along with XGBoost 2.0.3, scikit-learn 1.5.1 (random forest, AdaBoost, and gradient boosting), scikit-survival 0.23.0 (random survival forest), and SHAP 0.46.0 (model interpretability and feature importance analysis).

### Statistical Analysis

A multivariable Cox proportional hazards models was used to assess the contribution of preoperative SUVmax to postoperative recurrence. SUV_max_ was analyzed as both a continuous variable and a categorical variable in the separate models. The optimal cutoff value for categorizing SUV_max_ was determined using an ROC curve analysis, maximizing the Youden index. The AUC was used to assess the discriminative ability of the SUV_max_ in predicting recurrence. Other features of the ML model were also incorporated as potential confounders. This study included 149 recurrent cases, allowing up to approximately 15 variables in the model.^19^ Confounders included age, sex, pathologic stage, histologic type, Ly, v, NLR, PD-L1 expression, and adjuvant chemotherapy status. The proportional hazards assumption in the Cox models was assessed by examining Martingale residual plots. Multicollinearity among variables was evaluated using the variance inflation factor (VIF), with <5 indicating no multicollinearity.^20^ Analyses were conducted using Easy R (Saitama Medical Center, Saitama, Japan), with significance set at *P* < .05.^21^

### Ethical Approval Statement

This study was approved by the institutional review board (IRB) of the National Hospital Organization KCMC (approval number: 2023-73) and was carried out in accordance with the Declaration of Helsinki. The IRB of KCMC waived the requirement for informed consent from all research participants because of the retrospective and anonymous nature of the study. Information about opting out of this study is provided on the KCMC home page.

### Patient Consent Statement

Owing to the retrospective nature of this study, informed consent from the patients was not required.

## RESULTS

### Patient Characteristics

The study cohort consisted of 643 patients who were randomly stratified into the training (n = 514, 80%), validation (n = 64, 10%), and test (n = 65, 10%) sets. The incidence of postoperative recurrence was 23%, and 149 patients developed disease recurrence. The median preoperative SUV_max_ for the entire cohort was 3.6 (IQR, 1.3-8.8). The median patient age was 71 years (IQR, 65-76 years), and 59% of the patients were male. The most common histologic type was adenocarcinoma (ADC; n = 476, 74%), followed by squamous cell carcinoma (n = 115, 18%) and other subtypes (n = 52, 8%). The pathologic disease stages were classified as stage I (71%), stage II (17%), and stage III (12%). The median RFS was 1,047 (IQR, 529-1,471). There were no statistically significant differences in patient characteristics among the training, validation, and test sets, indicating a homogeneous distribution (Table 1).

**TABLE 1.** Clinical Characteristics and Outcomes of 643 Patients Undergoing Lung Cancer Resection

Characteristic	Total Cohort (N = 643)	Training Cohort (n = 514)	Validation Cohort (n = 64)	Test Cohort (n = 65)	*P*
Continuous variables, median (Q1-Q3)					
SUV_max_	3.6 (1.3-8.8)	3.5 (1.2-8.8)	3.9 (1.3-7.8)	3.7 (1.7-8.8)	.77
Age, years	71 (65-76)	71 (65-76)	70 (65-76)	71 (67-76)	.93
PD-L1 expression (TPS [%])	5 (0-36)	5 (0-30)	5 (0-50)	5 (0-55)	.67
NLR	1.8 (1.4-2.4)	1.8 (1.4-2.4)	1.7 (1.3-2.2)	1.9 (1.4-2.6)	.44
RFS, day	1,047 (529-1,471)	1,050 (508-1,466)	1,097 (588-1,418)	828 (549-1,639)	.94
Categorical variables, No. (%)					
No. of recurrences	149 (23)	127 (25)	10 (15)	12 (19)	.17
Male sex	378 (59)	302 (59)	39 (60)	34 (52)	.31
Histologic types, No. (%)					.36
ADC	476 (74)	385 (75)	47 (73)	44 (68)	
SCC	115 (18)	85 (17)	13 (20)	17 (26)	
Others^a^	52 (8)	44 (8)	4 (6)	4 (6)	
Pathologic stage, No. (%)					.75
Stage I	453 (71)	367 (71)	45 (69)	42 (69)	
Stage II	111 (17)	84 (16)	11 (17)	14 (20)	
Stage III	79 (12)	63 (12)	9 (14)	9 (11)	
v1, No. (%)	444 (69)	361 (70)	44 (69)	39 (60)	.24
Ly1, No. (%)	397 (62)	324 (63)	39 (61)	34 (52)	.24
Adjuvant chemotherapy, No. (%)	66 (10)	52 (10)	8 (13)	6 (9)	.80

Abbreviations: ADC, adenocarcinoma; Ly, lymphovascular invasion; NLR, neutrophil-to-lymphocyte ratio; RFS, recurrence-free survival; SCC, squamous cell carcinoma; SUV_max_, maximum standardized uptake value; TPS, tumor proportion score; v, vascular invasion.

a Histologic types excluding ADC and squamous cell carcinoma in non–small cell lung cancer are as follows: among 52 cases, 17 had pleomorphic carcinoma, 16 had large-cell neuroendocrine carcinoma, 12 had adenosquamous carcinoma, and seven had large-cell carcinoma.

### ROC Curve Analysis and Kaplan-Meier Survival Curves

An ROC curve was generated to determine the optimal cutoff value for SUV_max_ for predicting postoperative recurrence (Data Supplement, Fig S1). The AUC was 0.72, and a cutoff value of 3.3 was selected on the basis of the ROC curve analysis. Patients were then divided into two groups according to their preoperative SUV_max_ values: low (<3.3) and high (≥3.3). Kaplan-Meier curves were constructed to compare the probability of RFS between the two groups (Data Supplement, Fig S2). The analysis revealed that patients with high SUV_max_ (≥3.3) had significantly worse RFS than those with low SUV_max_ (<3.3; log-rank test; *P* < .001).

### Evaluation of ML Models

Feature correlations were visualized via a heatmap (Data Supplement, Fig S3). The intercategory correlation coefficients were all <0.7, indicating no strong correlations among the selected variables. The ML models, including random forest, gradient boosting, XGBoost, and AdaBoost, were trained using a training data set for postoperative recurrence prediction. The predictive performance of the various ML models was assessed using a validation data set (Table 2). The random forest model demonstrated the highest ROC AUC of 0.90 (95% CI, 0.82 to 0.97). The random forest model also exhibited the highest PR AUC (0.56; 95% CI, 0.44 to 0.68) among the four models. AdaBoost achieved the best F1 score (0.46; 95% CI, 0.34 to 0.58), whereas the random forest model had the lowest Brier score (0.11; 95% CI, 0.03 to 0.18), indicating superior calibration. Bootstrap evaluation using OOB samples validated the random forest model. The model showed consistent performance: ROC AUC 0.82 (95% CI, 0.77 to 0.86), PR AUC 0.48 (0.36 to 0.60), accuracy 0.76 (0.70 to 0.82), F1 score 0.42 (0.23 to 0.62), and Brier score 0.14 (0.13 to 0.16; Data Supplement, Table S2). O the basis of the overall performance metrics, the random forest model was selected as the final model for further evaluation using the test data set (Data Supplement, Table S3). The random forest model maintained its predictive performance in the test cohort, with an ROC AUC of 0.80 (95% CI, 0.71 to 0.90), PR AUC of 0.55 (95% CI, 0.43 to 0.67), accuracy of 0.85 (95% CI, 0.76 to 0.93), F1 score of 0.50 (95% CI, 0.38 to 0.62), and Brier score of 0.13 (95% CI, 0.04 to 0.21).

**TABLE 2.** Predictive Performance of Various ML Models in Identifying Postoperative Lung Cancer Recurrence in the Validation Cohort

ML Model	Assessment Metrics (95% CI)				
	ROC AUC	PR AUC	Accuracy	F1 Score	Brier Score
Random forest	0.90 (0.86 to 0.97)	0.56 (0.44 to 0.68)	0.81 (0.72 to 0.91)	0.40 (0.28 to 0.52)	0.11 (0.03 to 0.18)
Gradient boosting	0.83 (0.82 to 0.92)	0.44 (0.32 to 0.56)	0.84 (0.75 to 0.93)	0.58 (0.46 to 0.70)	0.13 (0.05 to 0.21)
XGBoost	0.82 (0.73 to 0.92)	0.49 (0.37 to 0.62)	0.83 (0.74 to 0.92)	0.35 (0.24 to 0.47)	0.12 (0.04 to 0.20)
Ada boosting	0.83 (0.74 to 0.92)	0.48 (0.36 to 0.60)	0.78 (0.68 to 0.88)	0.46 (0.34 to 0.58)	0.15 (0.06 to 0.24)

Abbreviations: ML, machine learning; PR, precision-recall; ROC, receiver operating characteristic; XGBoost, extreme gradient boosting.

### Importance and Dependence of Features in the Random Forest Model

**FIG 1.**
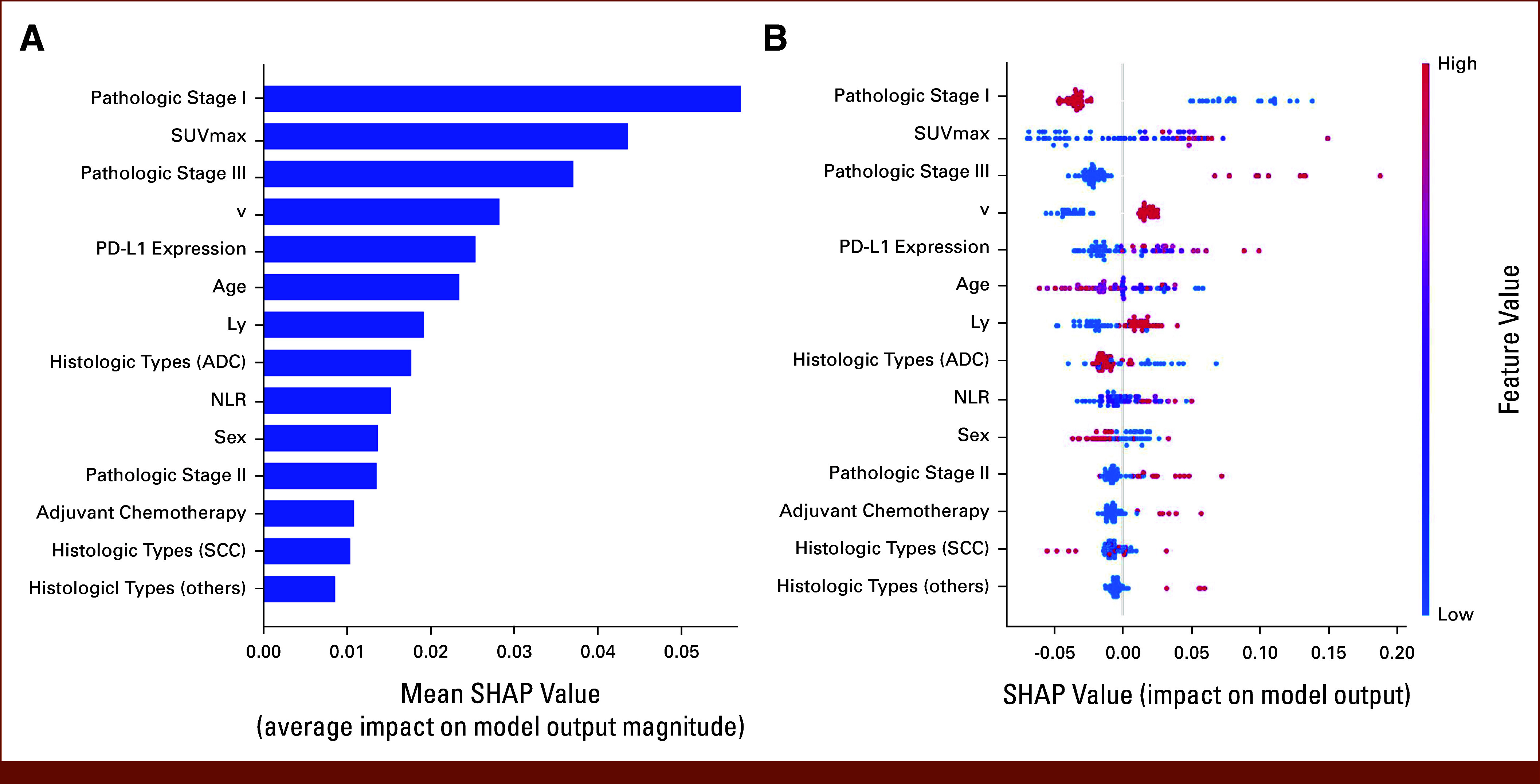
The SHAP analysis of the random forest model for predicting postoperative recurrence in resected NSCLC. (A) Feature importance ranking on the basis of the mean absolute SHAP values. Pathologic stage I has the highest impact on the model's prediction, followed by SUV_max_. (B) SHAP value distribution for each feature. Each dot represents an individual patient, with the color indicating the feature value (red for higher values and blue for lower values). The horizontal location of each dot shows the impact of that feature on the model's prediction for that patient. Dots to the right (positive SHAP values) indicate an increased probability of postoperative recurrence, while dots to the left (negative SHAP values) indicate a decreased probability. ADC, adenocarcinoma; Ly, lymphovascular invasion; NLR, neutrophil-to-lymphocyte ratio; NSCLC, non–small cell lung cancer; SCC, squamous cell carcinoma; SHAP, Shapley additive explanations; SUV_max_, maximum standardized uptake value; v, vascular invasion.

**FIG 2.**
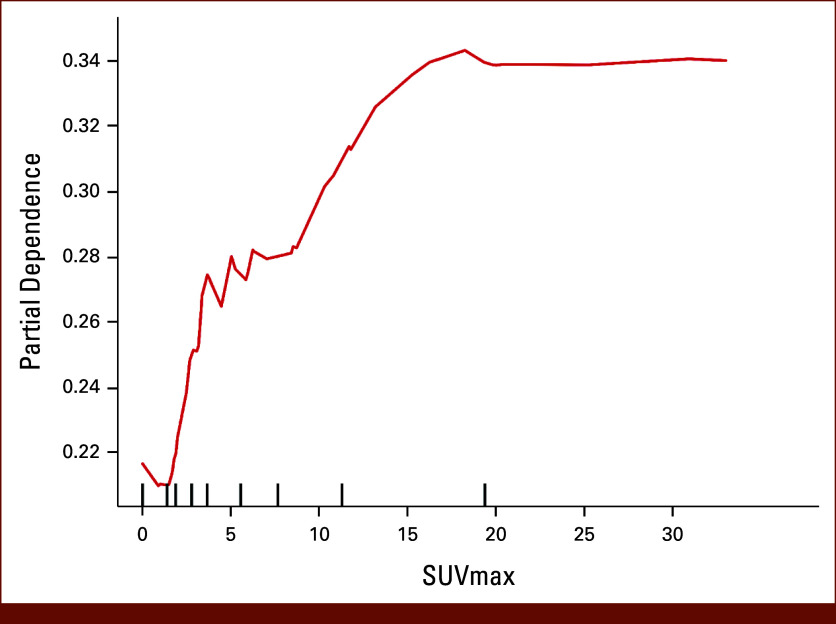
Partial dependence plot showing the relationship between SUV_max_ and the prediction of postoperative recurrence in NSCLC. The *x*-axis represents SUV_max_ values, while the *y*-axis shows the partial dependence, which indicates the contribution to the prediction of postoperative recurrence. NSCLC, non–small cell lung cancer; SUV_max_, maximum standardized uptake value.

The SHAP analysis using the random forest algorithm evaluated the importance of each feature in the postoperative recurrence prediction model. Pathologic stage I had the highest mean SHAP value, indicating its strong influence on model predictions (Fig 1A). Other influential features include SUV_max_ and pathologic stage III. The SHAP value distribution for SUV_max_ showed that higher values (red dots) were primarily concentrated in the positive SHAP value region, whereas lower values (blue dots) were predominantly in the negative region, suggesting that an increase in SUV_max_ was associated with an increased contribution to the model's prediction of postoperative recurrence (Fig 1B). The SHAP analysis quantified the impacts of categorical variables on the prediction of postoperative recurrence. Pathologic stage III was associated with the highest SHAP values. The non-ADC histologic subtypes showed higher positive SHAP values than ADCs. Adjuvant chemotherapy was associated with a positive SHAP values. The PDP revealed a nonlinear relationship between SUV_max_ and its influence on the model output, with a sharp increase in partial dependence in the SUV_max_ range of 2-5 and a more gradual increase beyond the value of 5 (Fig 2). The 10-fold cross-validation confirmed a robust nonlinear relationship between SUV_max_ and the risk of recurrence, with a sharp increase at 2-5 (Data Supplement, Fig S4). The PDPs for the expression of PD-L1, and NLR aligned with clinical observations (Data Supplement, Fig S5).

### Random Survival Forest Model for Predicting Postoperative Recurrence

**FIG 3.**
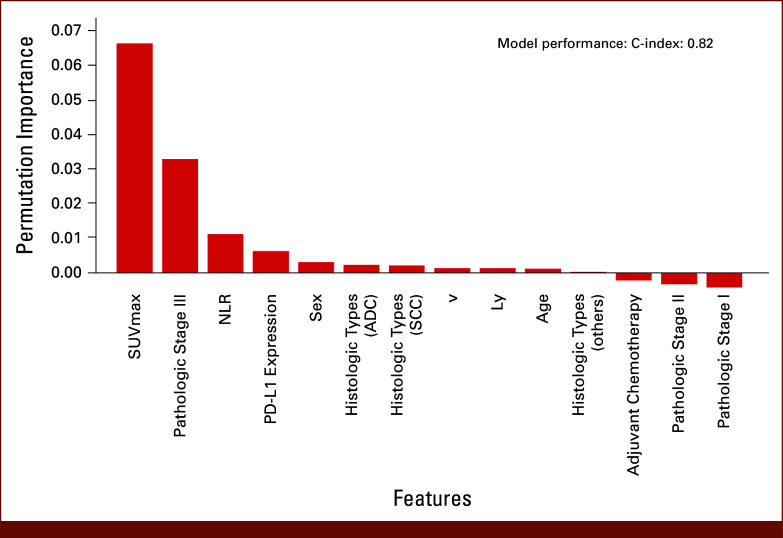
Permutation importance of features in predicting postoperative recurrence of NSCLC using a random survival forest model. The *x*-axis shows individual features, while the *y*-axis represents the permutation importance score. Higher scores indicate greater importance of the feature in the model's predictive performance. The model achieved a C-index of 0.82, demonstrating good discriminative ability. SUV_max_ emerged as the most important predictor. Features are arranged in descending order of importance from left to right. ADC, adenocarcinoma; C-index, concordance index; Ly, lymphovascular invasion; NLR, neutrophil-to-lymphocyte ratio; NSCLC, non–small cell lung cancer; SCC, squamous cell carcinoma; SUV_max_, maximum standardized uptake value; v, vascular invasion.

A random survival forest model was constructed using the same features as the random forest model. The hyperparameters were systematically optimized (Data Supplement, Table S1). The model achieved validation and test C-index of values 0.87 and 0.82, respectively. The feature importance was assessed using permutation importance (Fig 3). Permutation importance analysis revealed that SUV_max_ was the most influential feature, with a permutation importance score of 0.067.

### Multivariable Cox Proportional Hazards Analysis

A multivariable Cox proportional hazards model was used to assess the relationship between SUV_max_ and recurrence. Multicollinearity was ruled out (all VIF <5). The proportional hazards assumption was validated via Martingale residual plots, confirming the validity of the assumption (Data Supplement, Figs S6 and S7). In the multivariable analysis, an increase in SUV_max_ as a continuous variable was associated with a significantly elevated risk of postoperative recurrence (adjusted hazard ratio [HR], 1.03 [95% CI, 1.00 to 1.06]; Table 3; Data Supplement, Table S4). To illustrate the impact of SUV_max_ on recurrence risk, we plotted the theoretical HRs against the range of increase in SUV_max_ (Data Supplement, Fig S8). This exponential relationship, represented by the equation *y* = 1.03^*x*^, demonstrates that for every unit increase in SUV_max_, the HR for recurrence increases by a factor of 1.03. Furthermore, we explored the impact of SUV_max_ as a categorical variable using a cutoff value of 3.3 determined by ROC curve analysis. Patients with SUV_max_ ≥3.3 exhibited a significantly higher risk of postoperative recurrence than those with SUV_max_ <3.3 (adjusted HR, 2.32 [95% CI, 1.43 to 3.78]; Data Supplement, Table S5).

**TABLE 3.** Results of a Multivariable Cox Proportional Hazard Analysis of RFS According to the Value of SUV_max_

Variable	Unadjusted HR (95% CI), *P*	Adjusted HR (95% CI), *P*^a^
SUV_max_	1.07 (1.05 to 1.09), <.005	1.03 (1.00 to 1.06), .03

Abbreviations: HR, hazard ratio; NLR, neutrophil-to-lymphocyte ratio; RFS, recurrence-free survival; SUV_max_, maximum standardized uptake value.

a In the multivariable analysis, the HR was adjusted for age, the expression of PD-L1, NLR, sex, histologic type, pathologic stage, vascular invasion, lymphovascular invasion, and adjuvant chemotherapy.

## DISCUSSION

This study examined the relationship between the preoperative SUV_max_ and postoperative recurrence in patients with resected NSCLC. SHAP analyses across multiple models consistently identified SUV_max_ as an important predictor. PDP analysis revealed a nonlinear relationship, with the recurrence risk sharply increasing at SUV_max_ values of 2-5. Random survival forest and Cox models further confirmed the significance of SUV_max_. These findings across diverse methods suggest that SUV_max_ is a valuable biomarker for predicting the recurrence of NSCLC.

Several studies have reported an association between SUV_max_ and postoperative recurrence in NSCLC, demonstrating that patients with high SUV_max_ have a significantly increased risk of recurrence.^22-25^ The cutoff values for SUV_max_ in these studies are often 2-5, and the cutoff of 3.3 in this study is consistent with these reports. However, most previous studies analyzed SUV_max_ by dichotomizing on the basis of cutoffs, with a limited number of studies evaluating the continuous relationship between SUV_max_ and recurrence. To our knowledge, few studies have comprehensively assessed the importance of SUV_max_ using ML models in this specific context. The novelty of this study lies in its exploratory evaluation of the relationship between SUV_max_ and postoperative recurrence using both ML and statistical analysis. Although conventional statistical models showed an increasing risk of recurrence with increasing SUV_max_, the random forest model revealed a nonlinear relationship, with the impact of SUV_max_ on the prediction of recurrence sharply increasing at values of 2-5 and then gradually increasing thereafter. Bootstrap evaluation confirmed the high predictive accuracy of the RFS model. Ten-fold cross-validation consistently reproduced the nonlinear SUV_max_-recurrence risk relationship, with a characteristic increase at SUV_max_ values of 2-5. The PDP analysis in the random forest model accurately reproduced clinical observations; recurrence risk increased with higher PD-L1 expression and NLR values.^13,14^ These results affirm the model's robustness and the reliability of SUV_max_ as a recurrence predictor. This finding provides a novel and detailed understanding of the nonlinear relationship between the SUV_max_ and postoperative recurrence. Furthermore, the random survival forest model confirmed SUV_max_ as an important feature in survival time analysis. These results suggest the possibility of a more precise recurrence risk assessment using SUV_max_ and may contribute to personalized treatment for patients with NSCLC after surgery in clinical practice.

The SUV_max_ may be crucial in predicting postoperative recurrence in NSCLC, potentially reflecting the biological aggressiveness of the tumor. High SUV_max_ reflects increased tumor malignancy and proliferative potential,^26,27^ associated with GLUT upregulation.^28^ GLUTs facilitate glucose uptake, a key feature of the Warburg effect, characterized by enhanced glycolysis and lactate fermentation.^29^ Increased glucose uptake and glycolytic activity lead to genetic and epigenetic changes promoting tumor growth and invasion.^30^ Lactate production from the overexpression of GLUT and enhanced glycolysis acidifies the tumor microenvironment, promoting immunosuppression, tumor growth, metastasis, and drug resistance.^31,32^ Our study demonstrated a nonlinear relationship, with a sharp increase in the risk of recurrence even at relatively low SUV_max_ values, suggesting that the risk of recurrence rises from the early stages when tumor glucose metabolism increases. The upregulation of GLUT may be an early event in tumorigenesis, driving metabolic shifts and tumor progression. In addition, the SUV_max_ may reflect immune cell activity and immune checkpoint expression. Some studies have reported that metabolic reprogramming and an acidic microenvironment can impair immune cell function and upregulate the immune checkpoint expression, potentially leading to immunosuppression and tumor escape.^33,34^ The clinical significance of our study lies in demonstrating that SUV_max_, a noninvasive preoperative measure strongly reflecting both tumor aggressiveness and the tumor immune microenvironment, can be a powerful predictor of postoperative recurrence in NSCLC, with a sharp increase in risk, even at relatively low values. This finding may provide a valuable tool for stratifying patients and guiding perioperative treatment decisions, potentially improving outcomes through personalized management strategies.

This single-center retrospective observational study has limitations in terms of generalizability, particularly regarding potential differences in SUV_max_ values across institutions because of variations in PET scanner models and imaging protocols. Caution is necessary when generalizing the finding of a rapid increase in the risk of recurrence within the SUV_max_ range of 2-5. The retrospective study design also precluded the complete elimination of unknown confounding factors. Nevertheless, the discovered nonlinear relationship between SUV_max_ and recurrence risk may be relevant as a relative change pattern, even if absolute values differ between institutions. The observation that the risk of recurrence sharply increased even within relatively low SUV_max_ ranges suggests the potential clinical utility of SUV_max_ in the early assessment of the risk of recurrence. Multicenter studies for validation, standardization of PET-CT imaging protocols and imaging analyses, and the use of SUV ratios or change rates may be necessary to overcome these limitations. However, individual institutions can potentially construct recurrence risk assessment models using their own data, suggesting the feasibility of clinical application of SUV_max_ as a risk factor for recurrence at each institution, even before complete standardization is achieved.

Our findings suggest the potential clinical applications of SUV_max_ in the management of NSCLC, particularly in risk stratification and treatment planning. The nonlinear relationship between SUV_max_ and the risk of recurrence, especially at values 2-5, indicates its utility as an early risk indicator. Integrating SUV_max_ into preoperative decision making could guide management choices, including the need for neoadjuvant therapy or extent of surgical resection. Postoperatively, it may inform decisions regarding adjuvant therapy and surveillance strategies. Incorporating the preoperative SUV_max_ into risk assessment models may enhance treatment stratification, potentially facilitating more individualized therapeutic approaches. However, the clinical implementation of SUV_max_-based decision making requires validation through prospective, multicenter studies.

In conclusion, our analysis using ML and statistical approaches demonstrated that the preoperative SUV_max_ was a significant predictor of postoperative recurrence in resected NSCLC. The nonlinear relationship between SUV_max_ and recurrence risk, with a sharp increase at low values, suggests its potential as an early biomarker in high-risk patients. The importance of SUV_max_ across various analytical methods underscores its robustness as a prognostic factor. Incorporating SUV_max_ into clinical decision making could enhance risk stratification and personalized treatment strategies for NSCLC. Further research is required to validate these findings in diverse populations and settings.

## Data Availability

The database used in this study is not available to the public. Participants in our study did not provide consent for public sharing of their data. The Python code used in this study is available upon request from the corresponding author, k7kensuke@icloud.com.
